# Parliament and Publications

**Published:** 1911-09-16

**Authors:** 


### PARLIAMENT AND PUBLICATIONS.

### FAILURES OF MEDICAL PRACTITIONERS.

The number of failures among medical practitioners under Bankruptcy and Deeds of Arrangement Acts last year was rather less than in 1909. According to the official report by the Board of Trade, which has just been issued, the number of failures of "doctors of medicine, surgeons, &c.," in 1910 was 38, with total liabilities of £55,642; in 1909 the number of failures was 41, with liabilities of £74,216; in 1908 the number was 25, with £31,712 liabilities; in 1907 the number was 30, with £50,287 liabilities; and in 1906 31, with £34,672 liabilities.

### SIGHT TESTS IN THE MERCANTILE MARINE.

According to the report on sight tests used in Mercantile marine last year (Cd. 5876), the total number of candidates examined was 7,502; of these 7,393 candidates passed in form vision and 109 failed. In the colour vision tests, 7,252 candidates were successful and 141 failed; of the latter 69 were re-examined on appeal, of whom 29 passed and 40 failed. No case of failure to pass the colour ignorance test has been reported. The number of officers already in possession of certificates who, on coming up for examination in 1910, failed to pass the sight tests, was twenty. The whole question of the sight tests is now being carefully considered by a Departmental Committee appointed for the purpose.

### REPORT OF THE LOCAL GOVERNMENT BOARD FOR SCOTLAND.

The sixteenth annual report of the Local Government Board for Scotland deals with a variety of matters of medical interest. The number of local authorities that have extended the Infectious Diseases (Notification) Act, 1889, to include pulmonary phthisis continues to increase. At the close of 1910 thirty-five district local authorities (representing a population of 718,306) and forty-seven burghal local authorities (representing a population of 1,563,482) had obtained approval of the Board to the extension of the Act. The Notification of Births Act is now in force in twenty-eight districts. Doubt having been raised as to the procedure when an Inspector of Poor has afforded an old-age pensioner medical relief, the Board have expressed the view that the medical relief which may be granted to a pensioner without entailing forfeiture of the pension includes nursing provided on the recommendation of the medical officer. The Board have also stated that medical relief necessarily includes medicine and surgical appliances, and that the cost of these will rank as a charge against the medical relief grant. The number of samples examined in connection with the administration of the Sale of Food and Drugs Act in 1910 was 10,217, of which 1,265 were found to be adulterated or not up to standard. The report by the superintendent of the central vaccine institution for Scotland for the year 1910 states that the total number of tubes of lymph supplied in 1910 to parochial vaccinators and poorhouse authorities was 2,067, compared with 2,677 tubes in 1909. The substantial decrease in the number of tubes applied for would appear to be in some measure due to the increasing number of persons taking advantage of the exemption afforded to conscientious objectors by the Vaccination Act. During the last two years no applications have been received for humanised lymph, and the supplies issued have consisted entirely of glycerinated calf-lymph.—"Report of the Local Government Board for Scotland, 1910" (Cd. 5620), price 1s. 7d.

